# “Full factorial design of experiments dataset for parallel-connected lithium-ion cells imbalanced performance investigation”

**DOI:** 10.1016/j.dib.2024.110227

**Published:** 2024-02-22

**Authors:** Gabriele Piombo, Simone Fasolato, Robert Heymer, Marc F. Hidalgo, Mona Faraji Niri, Davide M. Raimondo, James Marco, Simona Onori

**Affiliations:** a Visiting Scholar at the Department of Energy Science and Engineering, Stanford University, USA; b WMG, University of Warwick, Coventry, United Kingdom; c Department of Electrical, Computer and Biomedical Engineering, University of Pavia, Pavia, Italy; d Department of Energy Science and Engineering, Stanford University, Stanford, USA

**Keywords:** Lithium-ion battery, Parallel-connected cells, Cell-to-cell parameters variation, Current and temperature imbalance, Design of experiments, Cell characterisation

## Abstract

This paper shares an experimental dataset of lithium-ion battery parallel-connected modules. The campaign, conducted at the Stanford Energy Control Laboratory, employs a comprehensive full factorial Design of Experiment methodology on ladder-configured parallel strings. A total of 54 test conditions were investigated under various operating temperatures, cell-to-cell interconnection resistance, cell chemistry, and aging levels. The module-level testing procedure involved Constant Current Constant Voltage (CC-CV) charging and Constant Current (CC) discharge. Beyond monitoring total module current and voltage, Hall sensors and thermocouples were employed to measure the signals from each individual cell to quantify both current and temperature distribution within each tested module configuration. Additionally, the dataset contains cell characterization data for every cell (i.e. NCA Samsung INR21700-50E and NMC LG-Chem INR21700-M50T) used in the module-level experiments. This dataset provides valuable resources for developing battery physics-based, empirical, and data-driven models at single cell and module level. Ultimately, it contributes to advance our understanding of how cell-to-cell heterogeneity propagates within the module and how that affects the overall system performance.

## Graphical abstract

**Figure:**
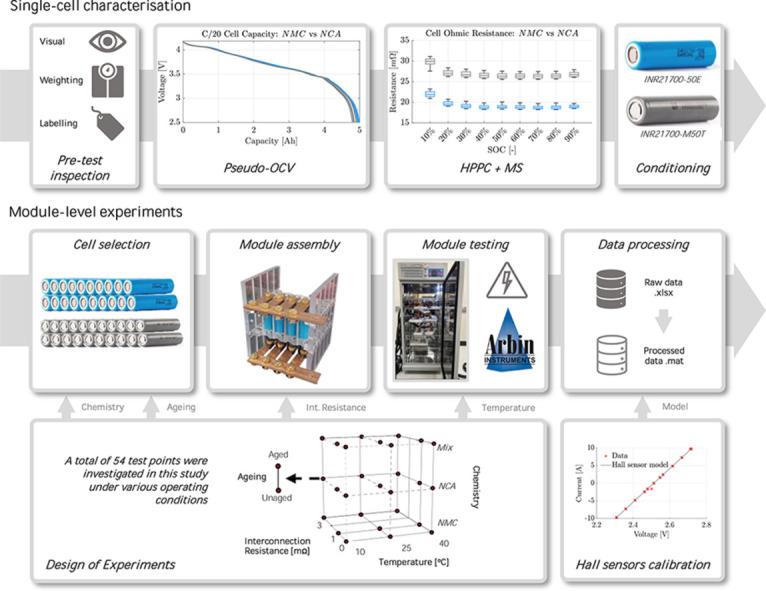

Specifications Table

**Table:** 

Subject	Electrical and Electronic Engineering.
Specific subject area	Design of Experiments (DoE) based campaign of parallel connected lithium-ion batteries and individual cells characterisation.
Data format	Raw and processed data.
Type of data	Tabulated Excel files (.xlsx) – Raw data MATLAB files (.mat) – Processed data
Data collection	**Parallel-connected Cells Performance Electrochemical Testing** A set of tests defined as (1) single cells characterisation and (2) module-level experiments. **(1) Single-cell characterisation** Discharge capacity is recorded via the Arbin LBT21024 system supplying a CC at a rate of C/20 to individual cells. Ohmic resistance is tracked at 10 % SoC intervals via hybrid pulse power characterisation (HPPC) and MultiSine test protocols. **(2) Module-level experiments** The Arbin Instruments LBT22013 system supplies a 3/4 C-rate discharge current profile to CC-CV fully charged parallel connected cells. The module terminals voltage, cells surface temperatures and parallel branch currents are measured via the Arbin system, Omega T-type thermocouples, and Honeywell Hall Effect SS495A calibrated sensors, respectively. The experimental temperature is maintained at the design target by the Amerex IC500R thermal chamber. The data is logged via MITS Pro Software at a sample rate of 1 s.
Data source location	Institution: Stanford Energy Control Laboratory, Energy Science and Engineering Department, Stanford University. City, State: Stanford, California. Country: United States of America. Latitude and longitude for collected samples/data: (37.426666918636386, −122.17397631867011).
Data accessibility	Repository name: *Parallel-connected module experimental campaign* DOI: 10.17632/zh58byr53c.1 Direct URL to data: https://data.mendeley.com/datasets/zh58byr53c/1
Related research article	Piombo, G., Fasolato, S., Heymer, R., Hidalgo, M., Faraji Niri, M., Onori, S., Marco, J.: “Unveiling the Performance Impact of Module Level Features on Parallel-Connected Lithium-Ion Cells via Explainable Machine Learning Techniques on a Full Factorial Design of Experiments”, Journal of Energy Storage 84, (2024), 110783, https://doi.org/10.1016/j.est.2024.110783.

## 1 Value of the Data

• Single-cell characterization, in the form of galvanostatic discharge, HPPC test, and MultiSine profiles are performed on a total of 39 single cells at 23 °C. In particular, the experimental campaign includes two fresh batches consisting of 18 Samsung INR21700-50E and 19 LG-Chem INR21700-M50T cells, aimed at identifying out-of-manufacture cell-to-cell variations. The third batch consists of one aged cell per chemistry type.
• The testing campaign for the parallel-connected battery modules are designed adopting a comprehensive full factorial DoE methodology. A total of 54 module-level experiments are conducted, considering four distinct factors within the DoE approach. These factors encompass 3 levels of testing: temperature (10, 25 and 40 °C), cell-to-cell interconnection resistance (0, 1 and 3 mΩ) and cell chemistry (all NMC, all NCA and mixed NMC/NCA), as well as two levels of cell ageing (aged and unaged).
• At the module level, the testing procedure consists of a CC-CV charging at a rate of C/3. The cut-off current of 50 mA is reached when holding at a CV of 4.2 V. Subsequently, a CC discharge is conducted at a rate of 0.75C. Throughout each test, alongside monitoring the overall module current and voltage, the currents delivered by each individual cell within the module, as well as their respective temperatures, were measured. The aim is to quantify the impact of cell-to-cell variations on module operations across a wide range of usage scenarios.
• The dataset provides parallel-connected module data that can enable the development of battery physics-based, empirical, and data-driven pack-level modelling frameworks for the understanding of cell-to-cell heterogeneity propagation. It also allows statistical analysis based on the DoE factors.
• To the best of the authors’ knowledge, this is the first dataset (that is both peer-reviewed and within the public domain) including data on cells connected in parallel, where the current and temperature of each cell connected in parallel are measured under different operating conditions and applying DoE methodology.

## 2 Background

The performed experimental campaign aimed to increase our level of understanding of the role of out-of-manufacture single-cell parameters distributions and module-level features on the uneven performance of parallel connected cells. Ultimately, the objective of this study is to inform about the mechanisms underlying the propagation of cell-to-cell variations, and how these variations ultimately influence the overall functioning of modules/packs. The dataset also encompasses standard characterisation test results for both unaged and aged batches of two distinct lithium-ion cell chemistries, offering an overview of the electrical properties distributions at the end of the manufacturing process. The developed dataset is associated with the publication in the Journal of Energy Storage [1] (https://doi.org/10.1016/j.est.2024.110783), which adds value to this article by providing an in-depth analysis via Explainable Machine Learning (XML) techniques. As the implemented methodology rigorously relied on a Full-Factorial DoE, this dataset can support further development of battery physics-based, empirical, and data-driven models at single cell and module level.

## 3 Data Description

**Fig. 1:**
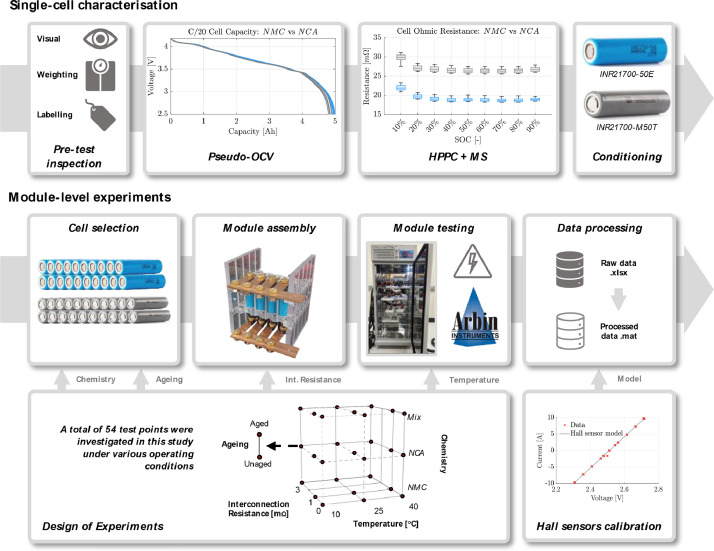
A visual flowchart of the implemented experimental procedures, selected steps, and design of experiments. Single-cell characterization (top) is performed before module-level testing (bottom) and consists of four phases: pre-inspection, namely visual investigation, weighting and sample labelling, identification of discharge capacity via the pseudo-OCV procedure, derivation of impedance via HPPC and MultiSine protocols, and conditioning for long-term storage. Module-level testing is grounded in the selected Full-Factorial DoE and consists of four phases: cells' selection and grouping, module assembly, test delivery, and data processing. Between the two campaigns, Hall-type current sensors are calibrated, and derived voltage-current maps are then leveraged to translate raw data into processed data.

The dataset includes two complementary experimental campaigns. The first one is a single cells’ characterisation campaign on all the 39 individual cells to identify sample properties and their out-of-manufacture distribution. The second one covers 0.75C CC discharge on four cells connected in parallel in a ladder configuration. To facilitate the reader's understanding of the testing procedures carried out in this study, a high-level visual flowchart of the steps involved in both campaigns is offered in Fig. 1. Cell characterisation was performed before the module-level tests. The 39 cells included in the study are 19 new LGM50T Lithium–Nickel–Manganese–Cobalt–Oxide (LiNiMnCoO_2_), 18 Samsung 50E Lithium Nickel–Cobalt–Aluminium Oxide Li(NiCoAl)O_2_, and one pre-aged cell for each chemistry. Both LG and Samsung cells implement Silicon-doped graphite (SiC) for the negative electrode. The technical specifications of the cells are included in Table 1 for completeness.

**Table 1:** Technical specifications of LG Chem INR21700-M50T [2] and Samsung INR21700-50E [3].

Manufacturer	LG Chem	Samsung
Model	INR21700-M50T	INR21700-50E
Positive electrode	Li(NiCoMn)O2	Li(NiCoAl)O2
Negative electrode	Graphite and Silicon	
Size (diameter × length)	21.44 × 70.80 mm	21.25 × 70.80 mm
Weight	69.25 g	69.00 g
Nominal capacity (Cn)	4.85Ah	4.90 Ah
Nominal voltage	3.63 V	
Charge cutoff voltage	4.2 V	
Discharge cutoff voltage	2.5 V	
Cutoff current (during CV)	50 mA	

Understanding the cell-to-cell variability is a crucial step to enhance the interpretability of module level imbalance phenomena. This is related to the impact of differences in cells’ properties have on the load and temperature distributions in parallel strings. Part of the experimental campaign is therefore focused on reference performance tests (RPT) on the cells’ used in the module-level experimental investigation described earlier in this paper. The list of characterisation steps involved is reported in Table 2. Every fresh cell is received from the manufacturer at voltages close to the nominal 3.6V. The two aged cells are long-term stored at 50 % SoC to reduce calendar ageing. The test cycle starts with a CC charge at a C/3 rate until 4.2V, followed by a CV phase terminating when the supplied current goes below 50 mA (Steps 1, 2). After a resting period of 60 min (Step 3), the cells’ discharge capacity is measured in Step 4 by applying current profiles with a C/20 rate until 2.5V limit is reached, obtaining Pseudo-Open Circuit Voltage (OCV) curves. Cells are then left resting for a period of 30 min (Step 5). The CC-CV charging procedure is repeated in Steps 6 and 7 up to 100 % SoC, followed by a 30 minutes rest in Step 8. To account for cells' internal properties dependency on SoC, the ohmic resistance measurements are performed at even intervals of 10 % by anticipating them with a constant current discharge profiles at a 1C rate (Step 9) followed by 60 minutes rest (Step 10). The ohmic resistance is sensed by means of two different protocols in Steps 11 and 12. First, HPPC current profiles are applied with a charge/discharge ratio of 0.75 and a duration of 10 s, as per automotive standards (Step 11) [4]. Then, MultiSine type dynamic current profiles are supplied following the procedure described in [5,6] with an α value of 0.6 and a pulse duration of 10 s (Step 12). A resting period of two minutes is imposed in Step 13 to allocate for cycle recursive management. Steps 9 to 13 are repeated until 2.5V is reached by applying multiple exit conditions. Last, a constant current constant voltage charge takes back the cells to 4.2V in Steps 14 and 15. The protocol ends when the supplied current goes below 50 mA. All the RPT are performed at a controlled thermal chamber temperature of 23 °C.

**Table 2:** Single cells characterisation campaign test protocol.

Step	Action	Exit condition
1	CC charge at 0.33 C-rate	4.2V reached
2	CV charge	Supplied current below 50 mA
3	Rest	60 min limit reached
4	CC discharge at 1/20 C-rate	2.5V reached
5	Rest	30 min limit reached
6	CC charge at 0.33 C-rate	4.2V reached
7	CV charge	Supplied current below 50 mA
8	Rest	30 min limit reached
9	CC discharge at 1 C-rate	6 min limit - to Step 10 or 2.5 V to Step 13
10	Rest	60 min limit reached
11	HPPC	10 s charge and 10 s discharge pulses
12	MS	Time-dependent current profile
13	Rest	2 minutes limit reached – to Step 9
14	CC	4.2V reached
15	CV	Supplied current below 50 mA

The module-level campaign aimed to enhance our level of understanding regarding the influence of various factors on the inconsistent performance of parallel connected cells. The considered factors can be referred, in part, to the single cell characterisation campaign, which provides valuable insights into the distribution of electrical properties. The remaining features refer to module-level characteristics, which serve as indicators of the influence exerted by design choices and operating conditions. A four-cells parallel string in a ladder configuration is tested, meaning the terminals of the module are connected on the same side. The experimental parameters encompass operating temperature, cell-to-cell interconnection resistance, chemistry (NCA, NMC, and mixed) and ageing status (Aged, and Unaged). The operating temperatures are 10 °C, 25 °C and 40 °C. The interconnection resistance levels are 0, 1 and 3 mΩ, leveraging high-precision shunt resistors (±1 %) soldered to 3.3 mm thick and 25.4 mm deep copper bars with negligible electrical resistance. The influence of the shunt soldering is tested upon manufacture and deemed to be negligible, as reported in [1]. In this study, a “Mixed” chemistry configuration refers to a combination of two NMC and two NCA cells in the parallel string being tested. The NCA cells are always kept closest to the terminals to represent the worst-case scenario of load imbalance among cells, as their ohmic resistance is lower than the NMC ones. On the other hand, the “NMC” and “NCA” configurations represent modules with cells of equal chemistry. Finally, the “Unaged” configuration denotes the connection of four fresh cells, while “Aged” refers to the inclusion in position 4 of one aged cell of the same chemistry. The location of the aged cell is selected to ensure a worst-case scenario standpoint in the form of maximum load imbalance, due to its higher ohmic resistance and lower discharge capacity when compared to unaged cells. The mixed and aged case includes a test where both the chemistries are mixed and two aged cells are included in the string. The factors ranges are selected upon literature and expert's review following previously conducted group campaigns on series-connected cells [7]. To mitigate the evolution of the cells’ characteristics over the experimental campaign, a randomized sampling methodology was developed. At each instance, four cells combination among the twenty in the batches are randomly selected and given a position (1–4) in the module to ensure repetitive tests on the same cells are minimized. The Stat-Ease Design Expert 22.0.2 software is employed to define the experimental design and entail the examination of all possible combinations of the factors and levels included resulting in a full-factorial DoE. The list of control variables is reported in Table 3, resulting in a total of 54 testing points.

**Table 3:** List of the control variables included in the DoE campaign.

Control variables	Levels	Unit
Interconnection Resistance	[0, 1, 3]	[mΩ]
Temperature	[10, 25, 40]	[°C]
Chemistry	[NMC, NCA, Mixed]	[-]
Ageing status	[Aged, Unaged]	[-]

**Fig. 2:**
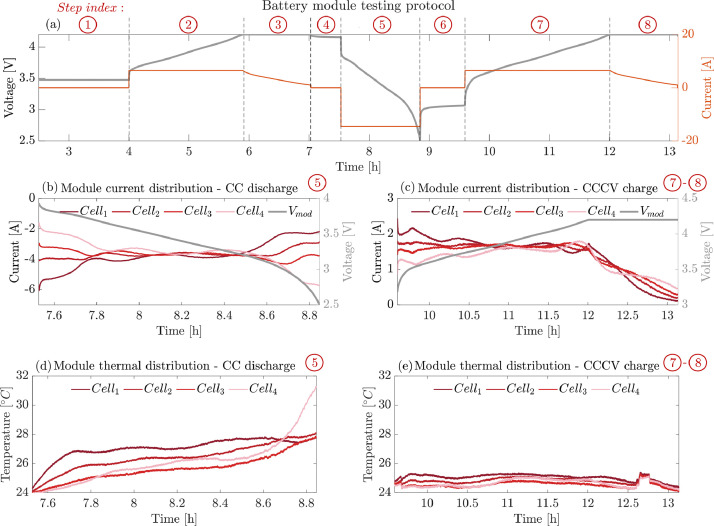
Cycling module-level testing protocol. (a) Terminal voltage (grey line) and total current (orange line) profiles across the 8 steps of the testing protocol listed in Table 3. (b and c) Distribution of the supplied current across the four parallel connected cells (red-scale lines) and module terminal voltage (grey line) during the constant-current discharge (Step ⑤) and CCCV charging (Steps⑦-⑧) phases. (d,e) Distribution of the individual cells’ surface temperatures during the constant-current discharge (Step ⑤) and CCCV charging (Steps ⑦-⑧) phases.

The module level test protocol consists of 8 consecutive steps, listed in Table 4 and illustrated in Fig. 2(a). A cycle starts with a 90 min resting period (Step ①) to allow the cells to self-balance and reach equilibrium (thermal and electrochemical) before testing, eliminating potential interferences due to cells' initial states. Steps ② and ③ comprise the charging phase of CC at C/3 up to 4.2V followed by a CV terminated when the module current (total supplied current) is lower than 200 mA. The module is then left to rest for 30 min (Step ④). Next, a 0.75C constant current discharge profile is applied until the terminal voltage reaches the lower limit of 2.5 V (Step ⑤). Self-balancing currents occur in the following one-hour rest phase (Step ⑥). The protocol is finalised by Steps ⑦ and ⑧ which are the repetition of Steps ② and ③ (a C/3 CC phase up to 4.2 V followed by a CV phase terminated when the supplied current goes below 200 mA) from a lower SoC level. The described procedure is repeated for each of the 54 testing points reported in Table 5.

**Table 4:** Module level campaign test protocol.

Step	Action	Exit condition
1	Rest	90 min limit reached
2	CC charge at 0.33 C-rate	4.2V reached
2	CV charge	Supplied current below 200 mA
4	Rest	30 min limit reached
5	CC discharge at a 0.75 C-rate	2.5V reached
6	Rest	60 min limit reached
7	CC charge at 0.33 C-rate	4.2V reached
8	CV charge	Supplied current below 200 mA

**Table 5:** Module-level campaign experimental design including tests’ order, factors’ specifications, cells’ position allocation in the module and links to the dataset repository files.

Test No.	Module No.	Chemistry	Ageing	R_Int_ [mΩ]	Temp. [°C]	Pos 1	Pos 2	Pos 3	Pos 4	Link to repository
1	1	NCA	Unaged	0	25	F7	F14	F15	F3	M1_NCA_UNAGED_0_25
2	1	Mixed	Aged	0	25	F18	GS3	P10	Y1	M1_Mixed_AGED_0_25
3	1	NMC	Aged	0	25	P4	P6	P2	Y1	M1_NMC_AGED_0_25
4	1	Mixed	Aged	3	25	F18	GS3	P3	Y1	M1_Mixed_AGED_3_25
5	1	NMC	Unaged	0	10	P13	P2	P6	P11	M1_NMC_UNAGED_0_10
6	2	Mixed	Unaged	3	10	F18	F16	P2	P14	M2_Mixed_UNAGED_3_10
7	1	NCA	Unaged	0	10	F17	F18	F10	F3	M1_NCA_UNAGED_0_10
8	2	NMC	Aged	1	10	P4	P6	P17	Y1	M2_NMC_AGED_1_10
9	1	NCA	Unaged	3	10	F17	F3	F15	F10	M1_NCA_UNAGED_3_10
10	2	Mixed	Aged	1	10	F18	GS3	P3	Y1	M2_Mixed_AGED_1_10
11	2	NCA	Unaged	1	25	F12	F2	F6	F10	M2_NCA_UNAGED_1_25
12	2	NCA	Aged	3	25	F11	F10	F4	GS3	M2_NCA_AGED_3_25
13	1	NMC	Unaged	3	10	P14	P5	P13	P6	M1_NMC_UNAGED_3_10
14	2	NCA	Aged	1	10	F9	F2	F4	GS3	M2_NCA_AGED_1_10
15	1	NMC	Aged	0	40	P4	P5	P18	Y1	M1_NMC_AGED_0_40
16	2	NCA	Unaged	1	40	F16	F12	F7	F5	M2_NCA_UNAGED_1_40
17	1	Mixed	Unaged	0	40	F8	F5	P17	P7	M1_Mixed_UNAGED_0_40
18	2	NMC	Aged	3	40	P12	P10	P2	Y1	M2_NMC_AGED_3_40
19	1	NCA	Aged	0	25	F5	F12	F9	GS3	M1_NCA_AGED_0_25
20	2	Mixed	Unaged	1	25	F7	F11	P12	P16	M2_Mixed_UNAGED_1_25
21	2	Mixed	Unaged	3	40	F5	F1	P15	P8	M2_Mixed_UNAGED_3_40
22	2	NCA	Unaged	3	40	F4	F5	F12	F9	M2_NCA_UNAGED_3_40
23	2	NMC	Unaged	1	40	P14	P3	P7	P12	M2_NMC_UNAGED_1_40
24	1	NCA	Aged	3	40	F12	F1	F16	GS3	M1_NCA_AGED_3_40
25	1	Mixed	Unaged	0	10	F13	F1	P5	P2	M1_Mixed_UNAGED_0_10
26	2	NMC	Aged	3	10	P3	P17	P15	Y1	M2_NMC_AGED_3_10
27	2	NMC	Aged	1	25	P19	P14	P3	Y1	M2_NMC_AGED_1_25
28	1	NMC	Unaged	3	25	P17	P4	P10	P16	M1_NMC_UNAGED_3_25
29	2	Mixed	Aged	1	40	F3	GS3	P7	Y1	M2_Mixed_AGED_1_40
30	1	NMC	Unaged	0	40	P14	P3	P8	P12	M1_NMC_UNAGED_0_40
31	1	Mixed	Unaged	0	25	F18	F12	P19	P14	M1_Mixed_UNAGED_0_25
32	2	NMC	Unaged	1	25	P20	P11	P17	Y1	M2_NMC_UNAGED_1_25
33	2	Mixed	Unaged	1	40	F2	F6	P11	P17	M2_Mixed_UNAGED_1_40
34	1	NCA	Aged	0	40	F15	F17	F3	GS3	M1_NCA_AGED_0_40
35	1	NMC	Unaged	3	40	P16	P9	P14	P5	M1_NMC_UNAGED_3_40
36	2	NCA	Aged	1	40	F13	F1	F5	GS3	M2_NCA_AGED_1_40
37	1	Mixed	Aged	0	10	F6	GS3	P19	Y1	M1_Mixed_AGED_0_10
38	2	Mixed	Aged	3	10	F5	GS3	P16	Y1	M2_Mixed_AGED_3_10
39	2	Mixed	Unaged	1	10	F3	F8	P17	P15	M2_Mixed_UNAGED_1_10
40	1	NMC	Aged	0	10	P19	P14	P16	Y1	M1_NMC_AGED_0_10
41	1	Mixed	Unaged	3	25	F17	F10	P14	P2	M1_Mixed_UNAGED_3_25
42	2	Mixed	Aged	1	25	F5	GS3	P8	Y1	M2_Mixed_AGED_1_25
43	1	Mixed	Aged	0	40	F7	GS3	P15	Y1	M1_Mixed_AGED_0_40
44	1	NMC	Aged	1	40	P10	P9	P16	Y1	M1_NMC_AGED_1_40
45	2	NCA	Unaged	1	10	F14	F5	F13	F12	M2_NCA_UNAGED_1_10
46	1	NCA	Aged	3	10	F18	F7	F11	GS3	M1_NCA_AGED_3_10
47	1	NCA	Unaged	3	25	F1	F3	F15	F13	M1_NCA_UNAGED_3_25
48	2	NCA	Aged	1	25	F6	F18	F8	GS3	M2_NCA_AGED_1_25
49	2	Mixed	Aged	3	40	F7	GS3	P11	Y1	M2_Mixed_AGED_3_40
50	1	NCA	Unaged	0	40	F1	F3	F9	F18	M1_NCA_UNAGED_0_40
51	1	NCA	Aged	0	10	F8	F12	F4	GS3	M1_NCA_AGED_0_10
52	2	NMC	Unaged	1	10	P2	P7	P12	P3	M2_NMC_UNAGED_1_10
53	1	NMC	Unaged	0	25	P7	P17	P19	P13	M1_NMC_UNAGED_0_25
54	2	NMC	Aged	3	25	P8	P14	P15	Y1	M2_NMC_AGED_3_25

**Fig. 3:**
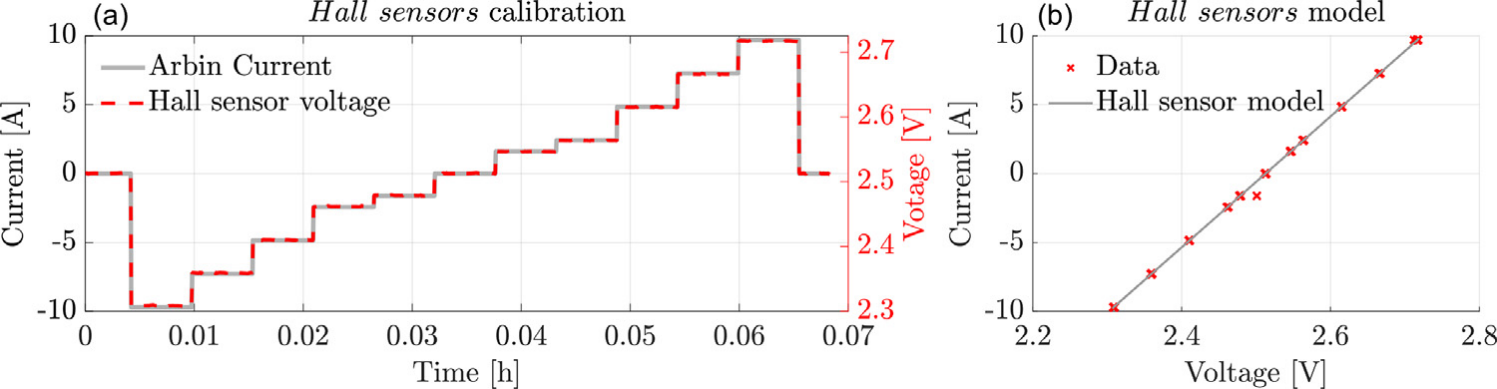
a) Hall sensors supplied-current ramp (grey line) to response voltage profile (red dashed line). b) Example of a resulting linear regression line (continuous grey) used to map the gathered voltage-current data (red crosses).

Throughout each test, alongside monitoring the overall module current and voltage (Fig. 2(a)), the currents delivered by each individual cell within the module and their respective temperatures were measured. Fig. 2(b)–(d) and Fig. 2(c)–(e) provide examples of the current and thermal distribution within the tested module during Step ⑤ and ⑦-⑧, respectively. Note that the current measurements on individual cells are gathered by means of Hall sensors. The Hall sensors working principle consists of the voltage across the output pins being linearly proportional to the magnetic field generated by the current source. The sensed voltage signals need coherently to be translated into currents via a calibration procedure. To calibrate the Hall sensors, a stepwise constant current profile is imposed via known current levels covering the C-rates range (−2C to 2C) used in the experimental campaign. The resulting profile is included in Fig. 3. Fig. 3(a) shows the correspondence between the supplied current and the sensed voltage. In Fig. 3(b), the linearity of the signal in the evaluated range is confirmed. The regression lines angular coefficients and biases are then derived to map each Hall sensor voltage to the actual current measurement. In total, 8 Hall sensors (i.e. four Hall sensors for each of the two manufactured module test benches) are calibrated and assigned to a specific string position before running the module-level campaign.

### 3.1 Dataset structure

The root folder containing all the files is named “Parallel-connected module experimental campaign” and is divided into the three sub-folders:

1. Single-cell characterisation
2. Hall sensor calibration
3. Module level experiments

**Fig. 4:**
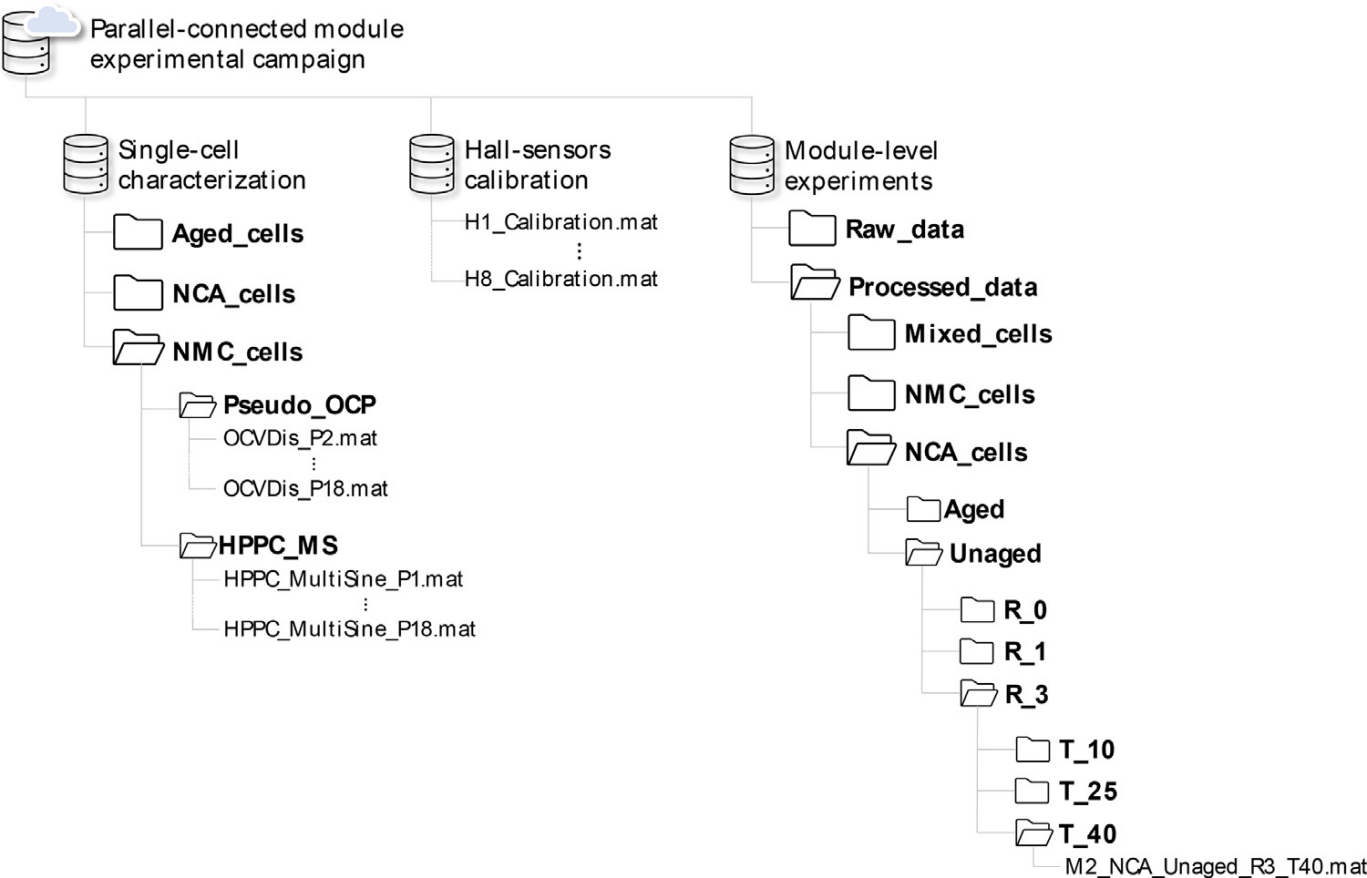
Dataset files structure.

An exhaustive description of each sub-folder is offered below, and schematically depicted in Fig. 4, to improve the dataset understanding and utilisation.

1. Single-cell characterisation

This folder contains the individual cells’ RPT campaign results including the data gathered from the NMC and NCA unaged and aged batches. Each of the “Aged_cells”, “NMC_cells” and “NCA_cells” sub-folders contain two folders named Pseudo OCV and HPPC-MS. These locations store the discharge Pseudo-OCV tests and HPPC-MultiSine results, respectively. The data was converted to the MATLAB format (.mat) and is named following the convention “TestType_CellName” where TestType can be either “OCVDis” or “HPPC_MultiSine” depending on the relevant test profile. “CellName” refers to the series and number given to each cell. NMC, NCA cells belong to the “P”, “F” series, respectively. NMC aged cell is coded as “Y1”, NCA one as “GS3”.

The .mat files contain double type arrays sensing:

• CurrentData: Supplied current profile in [A]
• OCV, VoltageData: Cell voltage profile in [V]
• TempData: Cell surface temperature profile in [°C]
• TimeData: Logged time profile in [s]

The “HPPC_MultiSine” .mat files also include two additional signals facilitating data handling via flags, namely:

• CycleIndex: Flag indicating the cycle number reached in the test protocol [-]
• StepIndex: Flag indicating the step number reached in the test protocol [-]

The Pseudo-OCV sampling time was set to 10[s]. The HPPC and MultiSine profiles are logged at a 1[s] sampling intervals in all phases apart from the pulses, where they are set to 0.1[s].

2. Hall sensors calibration

**Fig. 5:**
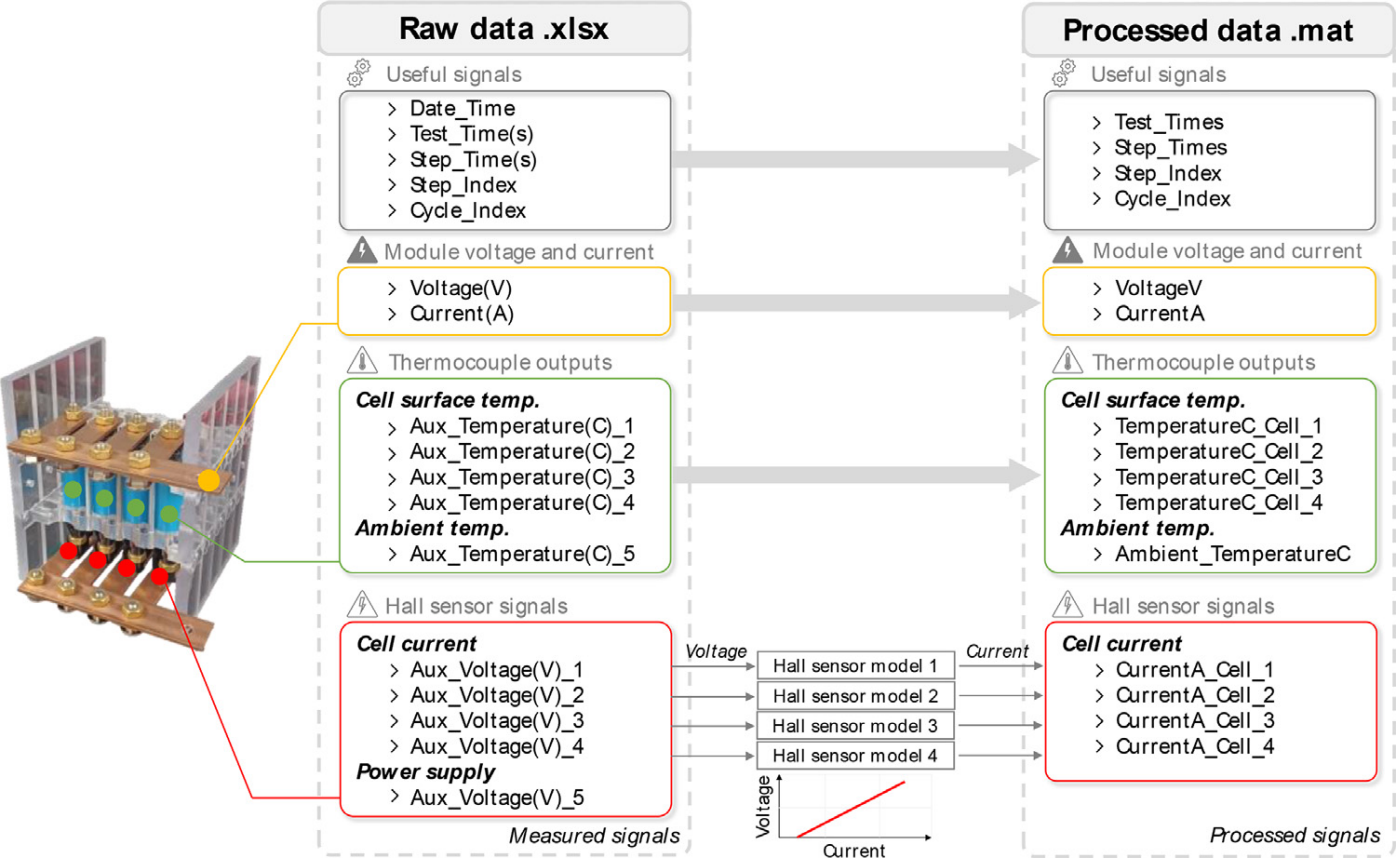
Comparison between module-level raw and processed data, where the signal names are highlighted.

This folder reports the data gathered while calibrating the voltage-current relationship used to derive the maps translating the raw to processed data in the module-level experimental campaign. A total of 8 Excel spreadsheets (.xlsx) logged files were converted to MATLAB (.mat) format to reduce storage space and are included following the naming convention “SensorNumber”_Calibration. To double the testing speed, a total of two twin modules as the one depicted in Fig. 5 were manufactured. Each module has four possible cell locations. Module number 1 (M1) includes locations 1-4, while module number two (M2) 5-8. Locations 1 and 5 are the closest to the module terminals, while 4 and 8 the furthest. Each sensor is logically allocated to a location and given the respective “SensorNumber”. The .mat files include a “Data” table, with headers referring to:

• Test_Times: Logged time profile in [s] from the test protocol start.
• Step_Times: Logged time profile in [s] resetting at each Step_Index increment.
• Step_Index: Flag indicating the step number reached in the test protocol [-].
• Cycle_Index: Flag indicating the cycle number reached in the test protocol [-].
• CurrentA: Supplied current profile in [A].
• HallVoltage: Hall sensor output voltage [V].
• PowerSupplyVoltage: Hall sensor power supply voltage output [V].
• CellTempData: Cell surface temperature profile in [°C].
• AmbientTempData: Thermal chamber ambient temperature profile in [°C].

Tracking the Hall sensor signal location is crucial as the regression lines depicted in Fig. 3 refer to the supplied current (“CurrentA”) and the Hall sensor voltage output (“HallVoltage”). The logging step used for sensors calibration is 0.1 [s].

3. Module-level experiments

This folder includes the data of the 54 test points module-level experimental campaign performed. To facilitate readability and usage, the data were allocated to sub-folders referring to the 4 features included in Table 3. The specific order of features is: chemistry, ageing, interconnection resistance and operating temperature, as reported in Fig. 4. The last sub-folder in the directory coherently includes individual test data named following the convention:

• “ModuleNumber_”: refers to which module was used to perform the test (M1, M2).
• “Chemistry_”: refers to the chemistry type of the tested cells (NMC, NCA, Mixed).
• “Ageing_”: refers to the ageing level of the tested cells (Aged, Unaged).
• “InterconnectionResistance_”: refers to the equipped busbar resistances (R0, R1, R3).
• “OperatingTemperature”: refers to the thermal chamber temperature (T10, T25, T40).

Both the “raw” and “processed” data are reported with respective main sub-folders. The raw data are saved as Excel spreadsheets with .xlsx type and refer to the data directly obtained from the MITS Pro software. The column headers refer to the sensed signal as follows:

• Date_Time: Logged testing time in “day/month/year hour:minutes:seconds” format.
• Test_Times: Logged time profile in [s] from the test protocol start.
• Step_Times: Logged time profile in [s] resetting at each Step_Index increment.
• Step_Index: Flag indicating the step number reached in the test protocol [-].
• Cycle_Index: Flag indicating the cycle number reached in the test protocol [-].
• VoltageV: Terminals voltage profile [V].
• CurrentA: Supplied module current profile in [A].
• Aux_Voltage(V)_(1-4): Hall sensors output voltage [V].
• Aux_Voltage(V)_5: Hall sensor power supply voltage output [V].
• TemperatureC_Cell(1-4): Cells surface temperature profile in [°C].
• Ambient_TemperatureC: Thermal chamber ambient temperature profile in [°C].

To allow for a fast data analysis, the raw data are converted to .mat type files and reported as “processed”. The data included were neither filtered nor resampled. Both raw and processed data share the same structure with the exception of the Hall sensors and power supply voltages (“Aux_Voltage_1-5”) which are converted to current profiles (Current(A)_”CellNumber”) leveraging the data included in the “Hall sensors calibration” folder. A schematic comparison of the data included in the raw and processed table data is offered in Fig. 5. The processed table therefore results in 15 columns, with a new header indicated as:

• CurrentA_Cell(1-4)

The “Data_processed” files contain an additional field indicated as “Cells_name”. The “Cells_name” field can be used to identify which cells were allocated to the individual tests and is composed by a table with four columns mapping the relative position in the module (headers) with the code of the cell taken from the “Single-cell characterisation” folder.

## 4 Experimental Design, Materials and Methods

**Fig. 6:**
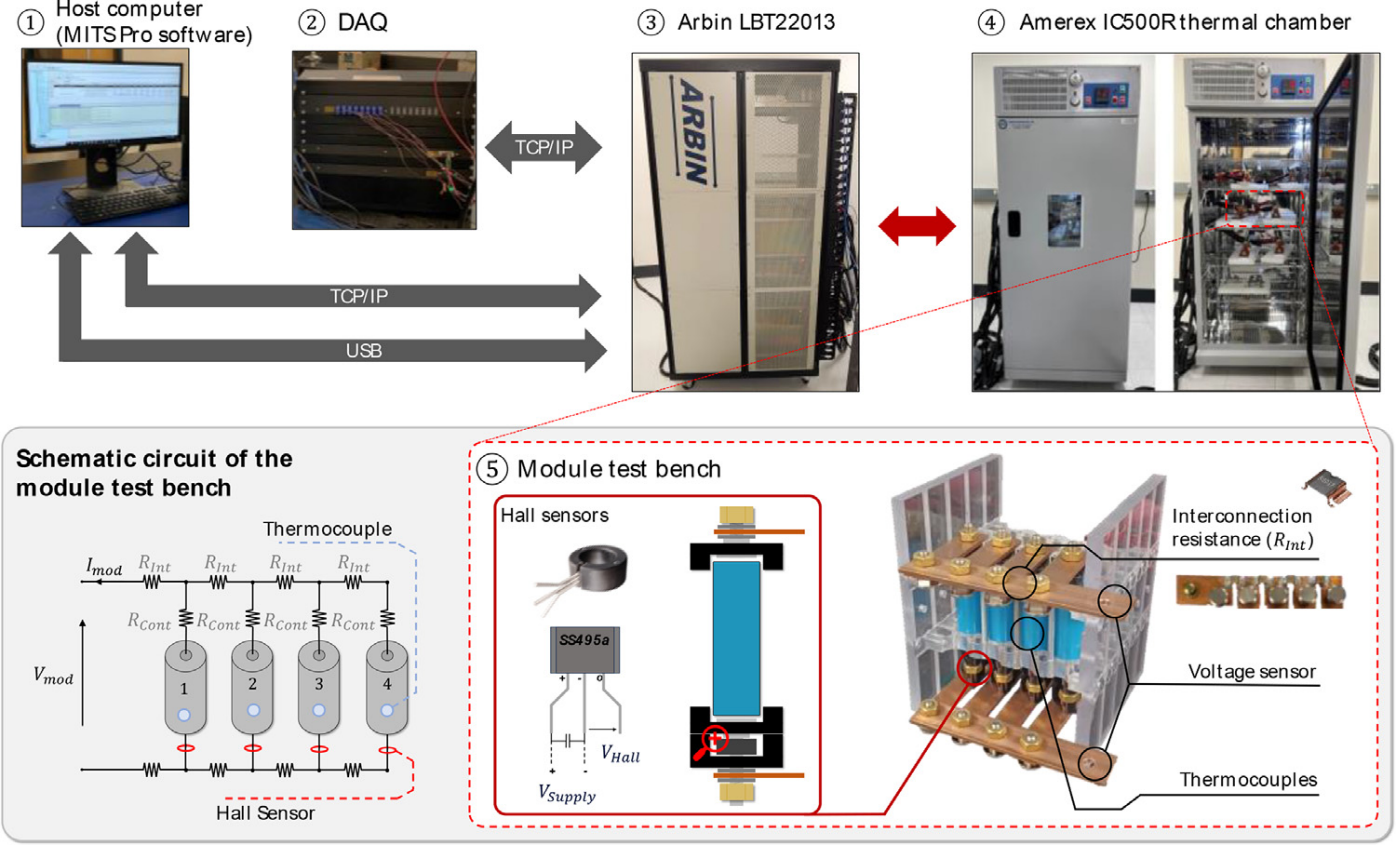
Equipment available at the Stanford Energy Control Lab [8].

The equipment available at the Stanford Energy Control Lab [8] and employed in the experimental campaign is shown in Fig. 6. The module cycling tests are designed with the MITS Pro software ①, which allows to define protocols, i.e., the sequence of steps to be followed to perform an experiment. To supply the battery module with the desired current profile and collect sensor data (i.e. module voltage, hall sensor voltages, and cell surface temperatures), the Arbin LBT22013 ③ is employed in conjunction with the Data Acquisition System (DAQ) ②. During testing, each battery module ⑤ is tested within the Amerex IC500R thermal chamber ④ and is instrumented with 5 T-type thermocouples placed to measure the surface temperatures at the centre of each cell, as well as the ambient temperature. Besides, four Honeywell SS495A Hall sensors are installed in each module to measure parallel paths currents. Hall-principle-based instruments were compared and selected over standard shunt resistors as the latter require a compromise between signal accuracy and influence on module's current distribution to be made. The larger the shunt resistance, the larger the voltage drop and hence signal-to-noise ratio. Nevertheless, the larger resistance mitigates the cell-to-cell ohmic resistance heterogeneity impact on current distribution, influencing the test results. Hall sensors operate via an external 5V circuit and hence do not present this limitation, despite requiring some measures to ensure an adequate setup accuracy. The Hall sensors are mechanically inserted and glued into ferrite rings to improve the signal-to-noise ratio and increase the reading scale. To prevent operators’ influence on sensor measurements, the ferrite rings are fixed around the current carrying connector at the negative terminal of each cell. In this way, the modules preparation does not require moving the sensors at each testing occurrence. Shielded cables are used to enclose signals and terminals legs are soldered and insulated to mechanically stabilize them and avoid shorts during operation. A 1mF capacitor is soldered across the 5V power supply to stabilize the input signal and mitigate its impact on readings. The raw data of each test is exported in Excel spreadsheets (.xlsx) file format. For more information about the setup the reader is referred to [9]. The Stat-Ease Design Expert software version 22.0.2 is used to develop the experimental design, factors levels and tests order.

## Limitations

Not applicable.

## Ethics Statement

The authors confirm that this work meets the ethical requirements of the journal.

## CRediT authorship contribution statement

**Gabriele Piombo:** Conceptualization, Methodology, Investigation, Data curation, Visualization, Writing – original draft. **Simone Fasolato:** Methodology, Investigation, Data curation, Visualization, Writing – original draft. **Robert Heymer:** Methodology. **Marc F. Hidalgo:** Methodology. **Mona Faraji Niri:** Methodology, Supervision, Writing – review & editing. **Davide M. Raimondo:** Supervision, Writing – review & editing. **James Marco:** Supervision, Writing – review & editing. **Simona Onori:** Supervision, Writing – review & editing.

## Data Availability

Full factorial design of experiments dataset for parallel-connected lithium-ion cells imbalanced performance investigation (Original data) (Mendeley Data).
